# High accuracy in detecting HER2‐low status in FNA of primary and metastatic breast cancer

**DOI:** 10.1002/cncy.70085

**Published:** 2026-02-27

**Authors:** Giuseppe D’Abbronzo, Stefano Lucà, Immacolata Cozzolino, Marina Accardo, Carminia Della Corte, Francesco Iovino, Simona Parisi, Ilaria Tedesco, Francesco Ingallinella, Francesca Grasso, Renato Franco, Marco Montella

**Affiliations:** ^1^ Department of Mental Health and Physic and Preventive Medicine University of Campania Luigi Vanvitelli Naples Italy; ^2^ Caserta Local Health Authority Caserta Italy; ^3^ Department of Precision Medicine Faculty of Medicine and Surgery University of Campania “L. Vanvitelli” Naples Italy; ^4^ Department of Advanced Medical and Surgical Sciences University of Campania “Luigi Vanvitelli” Naples Italy

**Keywords:** cell block, diagnostic accuracy, fine‐needle aspiration cytology, HER2‐low breast cancer, immunocytochemistry

## Abstract

**Background:**

HER2‐positive invasive breast carcinomas (IBCs) account for 15% of breast cancers and are driven by ERBB2 gene amplification. Although historically associated with aggressive behavior, HER2‐targeted therapies have significantly improved outcomes. HER2 status is routinely assessed by immunohistochemistry (IHC) and in situ hybridization (ISH). Recently, tumors with low HER2 expression (IHC 1+ or 2+ without amplification), previously classified as HER2‐negative, have emerged as a clinically relevant subgroup. Fine‐needle aspiration cytology (FNAC) is a minimally invasive alternative to core needle biopsy, particularly useful in inoperable or metastatic settings. FNAC‐derived cell blocks (CBs) allow immunocytochemical (ICC) evaluation of biomarkers.

**Methods:**

This retrospective study included 46 FNAC‐derived CBs from breast cancers (34 primary tumors and 12 metastases) collected at Vanvitelli University Hospital. ICC evaluation of HER2, estrogen receptor (ER), and progesterone receptor (PR) was independently performed by two expert pathologists and compared with corresponding histological assessments. Diagnostic performance was evaluated using sensitivity, specificity, predictive values, concordance rates, and receiver operating characteristic (ROC) curve analysis.

**Results:**

ICC on FNAC‐derived CBs showed good diagnostic performance for HER2‐low tumors. Sensitivity ranged from 56.3% to 59.4%, whereas specificity was high (85.7%–92.9%). Positive predictive values reached 90.0%–95.0%, whereas negative predictive values were lower (46.2%–50.0%). Concordance between cytological and histological HER2‐low assessment exceeded 90%, with ROC area under the curve values of 0.71–0.76. ER showed excellent concordance, whereas PR demonstrated moderate agreement.

**Conclusions:**

FNAC‐derived CBs are a reliable tool for identifying HER2‐low breast carcinomas when histological samples are unavailable or limited, emphasizing the need for standardized evaluation criteria.

## Abstract 2

This study demonstrates that fine‐needle aspiration cytology with cell block preparation provides high specificity and positive predictive value for the detection of HER2‐low breast carcinomas. Despite moderate sensitivity, cytological assessment represents a valuable diagnostic alternative when core biopsy is unavailable or impractical.

## INTRODUCTION

HER2‐positive invasive breast carcinomas (IBCs) are clinically significant subtypes, accounting for approximately 15% of all breast cancer cases. HER2 overexpression is driven by ERBB2 gene amplification, commonly seen in two molecular subtypes of breast cancer characterized by aggressive tumor behavior. The introduction of HER2‐targeted therapies, however, has transformed the treatment landscape for these tumors, leading to substantial survival benefits in both early‐stage and metastatic settings.^1^ HER2‐positive status is now determined through evidence of protein overexpression, scored as 3+ by immunohistochemistry (IHC), or by detecting gene amplification using in situ hybridization (ISH) assays.^2^ In cases with an equivocal IHC score of 2+, confirmatory ISH testing is required to ascertain HER2 status. Historically, tumors scoring 0 or 1+ on IHC, or those with a 2+ score and negative ISH findings were classified as HER2‐negative, effectively excluding these patients from HER2‐targeted treatment options. Recently, this classification system has undergone significant re‐evaluation. In fact, pivotal trials, such as DESTINY‐Breast 04, have shown that innovative anti‐HER2 agents can extend therapeutic benefits to patients with low HER2 expression, traditionally included in the HER2‐negative class.^3^ Indeed, cancers with low HER2 protein expression have emerged as a clinically relevant subgroup, prompting interest in expanding treatment options for these patients.^3^, ^4^, ^5^ Specifically, breast cancers with HER2 IHC scores of 1+ or 2+ and negative ISH findings are now classified under the proposed term “HER2‐low breast cancer.”^6^, ^7^ To aid in identifying such cases, the 2023 American Society of Clinical Oncology/College of American Pathologists (ASCO/CAP) guidelines recommend enhanced evaluation procedures, such as tissue evaluation at 40× microscope magnification to detect faint or focal HER2 expression, possible second opinions for borderline cases and the use of controls with a range of HER2 expression levels.^6^ However, distinguishing HER2‐low cases from HER2‐negative remains a challenging task because of variability in inter‐pathologist concordance, pre‐analytical processing factors, and intratumoral heterogeneity.^8^

In routine practice, percutaneous core needle biopsy (percutaneous‐CNB) is the preferred procedure for the diagnosis of breast cancer, but its feasibility can be limited by several factors, including patient inoperability, refusal to consent to an invasive procedure, or the unavailability of CNB in peripheral hospitals and resource‐limited settings.^9^ Thus, fine‐needle aspiration cytology (FNAC) offers a more cost‐effective and less invasive alternative to CNB for obtaining diagnostic material essential for guiding clinical decision‐making, monitoring, and treatment planning.^10^ In addition, when specific metastatic sites are difficult to access with CNB, FNAC can be easily performed, being a less invasive and better‐tolerated procedure. Indeed, it is estimated that approximately 30% of patients with advanced cancer cannot undergo biopsy or provide samples that meet quality control standards due to factors such as insufficient material and low tumor cellularity. In these specific settings, FNAC often serves as a reliable and minimally invasive alternative.^11^ Furthermore, the decalcification process required for biopsies of bone metastases may affect the interpretative accuracy of HER2 evaluation. Therefore, the cell block (CB) obtained from FNAC of bone metastases, which does not require decalcification, can represent a valuable tool for HER2 assessment.^12^

In our study, we retrospectively evaluated HER2‐low status in CBs obtained from cytological samples of breast cancer or metastases, comparing them with the corresponding histological samples, considered the gold standard for such detection, to assess the diagnostic accuracy of HER2 status in cytology and evaluate its possible applicability in clinical practice.

## MATERIALS AND METHODS

### Selection of cases

Patients with primary or metastatic invasive breast carcinoma diagnosed by FNAC were retrospectively identified from the archives of Vanvitelli University Hospital. A total of 74 FNAC‐derived CBs were initially collected.

Twenty‐eight cases were excluded due to inadequate material for reliable immunocytochemical evaluation, including insufficient tumor cellularity (<100 representative neoplastic cells), suboptimal CB preservation, or lack of matched histological samples available for comparison.

The final study cohort therefore consisted of 46 FNAC‐derived CBs from primary (34 cases) and metastatic (12 cases) breast carcinomas. Inclusion criteria for the final cohort were: 1) cytological diagnosis of breast carcinoma; 2) availability of an FNAC‐derived CB containing at least 100 representative tumor cells and suitable for immunocytochemical evaluation of estrogen receptor (ER), progesterone receptor (PR), and HER2 (scores 0, 1+, or 2+ without gene amplification); and (3) availability of matched histological samples assessed by immunohistochemistry for ER, PR, and HER2, which served as the reference standard.

In cytological samples, the distinction between ductal carcinoma in situ and invasive carcinoma was not attempted due to the intrinsic limitations of cytology in evaluating architectural features. The definitive diagnosis of invasive carcinoma was established on the corresponding histological samples.

#### FNAC procedure and sample processing

In all cases, an interventional cytopathologist performed Ultra‐Sound (US)‐guided FNAC, with rapid on‐site evaluation conducted on air‐dried smears stained with Diff‐Quik to assess sample adequacy. If the material was inadequate or insufficient, a second pass was performed. Additional direct smears were alcohol‐fixed and stained with Papanicolaou. A further pass was collected in 5 mL of (unbuffered) formalin for CB preparation. CBs were processed using the Epredia Shandon Cytoblock Cell Block Preparation System, following the manufacturer’s protocol: after fixation, cells were concentrated by centrifugation, the supernatant removed, the cellular pellet mixed with the Cytoblock gel reagents, transferred into a Cytoblock cassette mounted on a Cytoclip, and cytocentrifuged in a Cytospin at 1500 rpm for 5 minutes (low acceleration). The resulting “cell‐button” was overlaid with another drop of gel, the cassette closed, then fixed in unbuffered formalin (e.g., Formal‐Fixx) and routinely processed for paraffin embedding. Sections from the CB were used for immunocytochemistry (ICC) for ER, Progesteron Receptor (PgR), and HER2. Histological samples—whether from primary or metastatic carcinoma—were fixed in 10% neutral buffered formalin for 6 to 48 hours, processed in the usual way for paraffin embedding, and analyzed by immunohistochemistry (IHC).^13^ Histological samples, regardless of whether the material was obtained from primary or metastatic carcinomas, were fixed in 10% neutral buffered formalin for at least 6 hours and up to 48 hours.

Histological samples, regardless of whether the material was obtained from primary or metastatic carcinomas, were fixed in 10% neutral buffered formalin for at least 6 hours and up to 48 hours in accordance with ASCO/CAP guidelines, to ensure standardized pre‐analytical conditions.

#### ICC and IHC procedure

**FIGURE 1:**
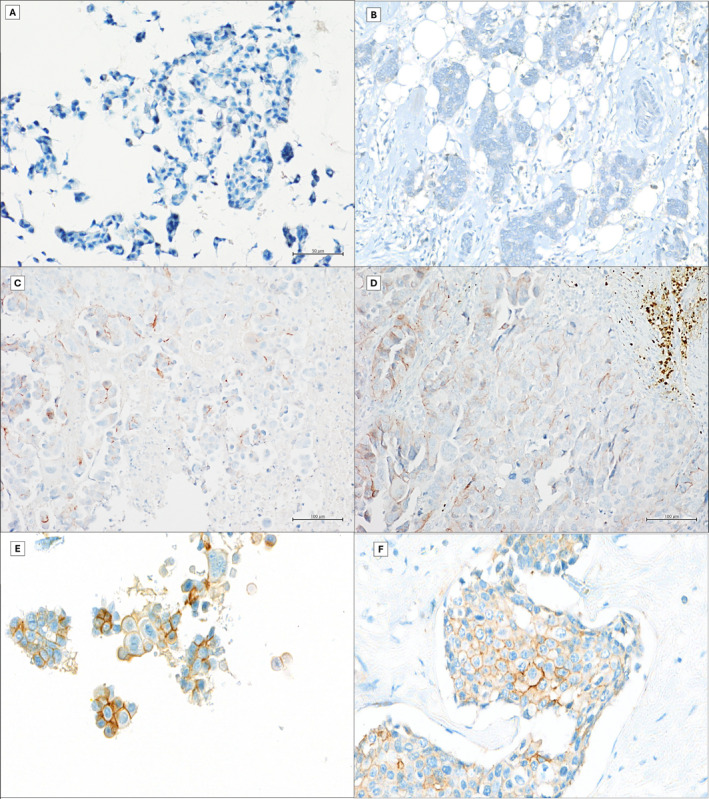
Comparison between cytological and histological HER2 evaluation (IHC DAB staining, magnification 40×): (A and B) HER2 0; (C and D) HER2 1+; and (E and F) HER2 2+.

**FIGURE 2:**
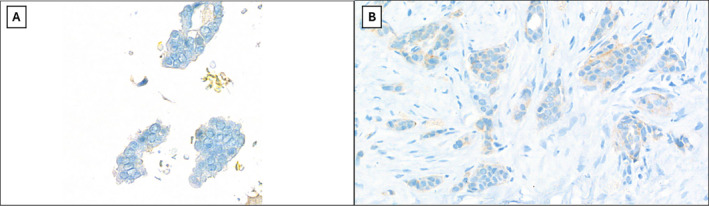
Example of a case with discrepancy in HER2 scoring between cytology and histology with a reclassification from score 0 in cytology (A) to 1+ in histology (B) (IHC DAB staining, magnification 40×).

The adequate CBs obtained from FNAC were immunostained for ER (rabbit monoclonal primary antibody, clone Sp1), PgR (rabbit monoclonal primary antibody, clone 1E2), and HER2 (rabbit monoclonal primary antibody, clone 4B5). ICC was performed on a Ventana platform (BenchMark ULTRA system, Ventana) according to the manufacturer's instructions.^14^ Similarly, IHC was performed on significant tissue blocks from surgical tumor samples. In both ICC and IHC, endometrial and breast carcinoma tissue sections were used as positive controls, whereas normal breast tissue, when present, served as an internal control. Nuclear staining in at least 1% of tumor cells was considered positive. The ICC results for ER and PR were classified as positive (>10% of cells showing positive nuclear staining) or negative.^15^ HER2 ICC results were interpreted as negative, 1+, 2+, or 3+ based on the complete membrane positivity and intensity of staining, along with the percentage of positive cells (Figure 1).^16^ Aberrant patterns, such as diffuse cytoplasmic, granular cytoplasmic, basal membrane‐like, luminal‐only, or extracellular staining, were considered nonspecific and were classified as negative (score 0). For each case, original data of HER2 expression were reported, but all cases were reviewed by two expert pathologists (R.F. and M.M.), observing CB and tissue samples at 40× microscope magnification (Figure 1, 2).

#### Fluorescence in situ hybridization

Fluorescence in situ hybridization (FISH) for HER2 amplification was performed using the PathVysion HER2 DNA probe kit (Abbott Molecular, Downers Grove, Illinois) as a reference standard in cases with equivocal IHC HER2 results, following the manufacturer's instructions. A total of 30 cells were analyzed in both CB and surgical specimens, and the ratio of HER2 to chromosome enumeration probe 17 (CEP‐17) was calculated. HER2 amplification was defined as a HER2/CEP‐17 ratio >2.2. A ratio <1.8 was considered negative, whereas a ratio between 1.8 and 2.2 was interpreted as equivocal.^17^

#### Statistical analysis

**FIGURE 3:**
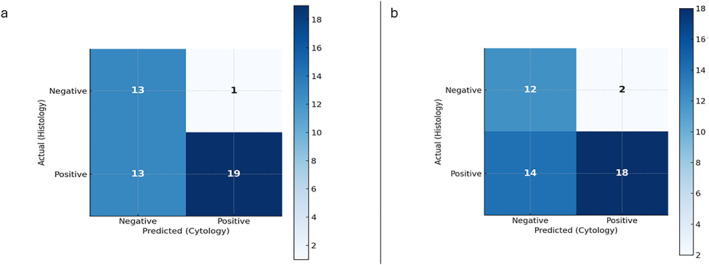
Confusion matrix HER2 histology versus HER2 cytology evaluation. (A) First pathologist. (B) Second pathologist.

Cytological and histological assessments were dichotomized as negative (ER and PgR <10%; HER2 score 0) or positive (ER and PgR >10%; HER2 scores 1+ or 2+). First, the agreement between cytological and histological assessment of HER2 was evaluated in absolute numbers both in original and in the revised sets. To determine the diagnostic value of cytology, statistical analysis was performed for HER2 expression on the revised sets for both the first and second pathologists. First, a confusion matrix was established to categorize true positives (TP), true negatives (TN), false positives (FP), and false negatives (FN) (Figure 3). These values were used to calculate the performance metrics specified in Tables 4 and 5:

- Sensitivity: the proportion of positive histologic cases (1+ or 2+) that were accurately diagnosed using cytology.
- Specificity: the proportion of cases that were histologically negative (score 0) and were accurately diagnosed using cytology.
- Positive predictive value (PPV): the probability that a positive cytological diagnosis will have a positive histological diagnosis.
- Negative predictive value (NPV): the probability that a negative cytological diagnosis will have a corresponding negative histological diagnosis.

**FIGURE 4:**
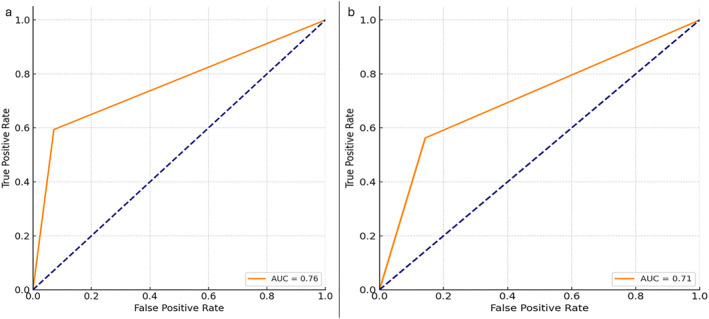
Receiver operating characteristic analysis: HER2 histology versus HER2 cytology evaluation. (A) First pathologist. (B) Second pathologist.

Moreover, we analyzed the cytology scoring diagnostic accuracy with respect to the histopathology report using the receiver operating characteristic (ROC) curve and area under the curve (AUC) for measuring the performance, or accuracy, of the test (Figure 4). Finally, the agreement between cytological and histological evaluation was also assessed for ER and PR using Cohen's κ. All statistical analyses were performed using Python (Scikit‐learn, Seaborn, and Matplotlib libraries).

## RESULTS

### Clinicopathological features

The clinicopathological features of our series are reported in Table 1. We included in the study 46 female patients with an average age of 62 years. Primary breast cancer was diagnosed in 34 of 46 cases (73.9%), whereas diagnosis of metastatic disease was made in 12 patients (12 of 46, 26.1%), with involvement of axillary lymph nodes in seven cases (15.2%) and the parietal pleura in five cases (10.9%). Concerning histological subtypes, our sample consisted of invasive breast carcinoma of no special type (IBC‐NST) in most cases (40 of 46, 87.0%),whereas the remaining cases were classified as follows: three invasive lobular carcinomas, one invasive papillary carcinoma, one mucinous carcinoma, and one metaplastic carcinoma. Finally, most of the IBCs were classified as luminal A (9 of 46, 19.6%) or luminal B (31 of 46, 67.4%), with only six (13.0%) cases of the triple‐negative type.

**TABLE 1:** Clinicopathological features of the series.

Clinicopathological features	No. of cases (%)
Age, years	
>65	18 (39.1)
<65	28 (60.9)
Tumor site	
Breast	34 (73.9)
Lymph node	7 (15.2)
Pleural	5 (10.9)
Primary breast cancer site	
Right breast	25
Right breast, upper outer quadrant	13
Right breast, lower outer quadrant	4
Right breast, upper inner quadrant	7
Right breast, lower inner quadrant	1
Left breast	21
Left breast, upper outer quadrant	12
Left breast, lower outer quadrant	2
Left breast, upper inner quadrant	7
Breast cancer histotype	
IBC‐NST	40 (86.9)
ILC	3 (6.5)
IPC	1 (2.2)
MuC	1 (2.2)
MeC	1 (2.2)

Abbreviations: IBC‐NST, invasive breast carcinoma of no special type; ILC, invasive lobular carcinoma; IPC, invasive papillary carcinoma; MeC, metaplastic carcinoma; MuC, mucinous carcinoma.

### HER2 expression status in cytological and histological samples

In the original evaluation, HER2 assessment on FNAC‐derived cell blocks showed a variable concordance with matched histological samples across the different ICC score categories (Table 2). Overall agreement between cytology and histology was limited for individual scores (0, 1+, and 2+), whereas concordance was higher when cases were grouped according to HER2‐low status. Specifically, when HER2 scores 0 and 1+ were considered together, concordance between cytological and histological evaluation reached 81.2%.

**TABLE 2:** Evaluation of HER2 expression status in cytology and histology samples performed in the initial diagnosis and in the revision sets (first and second pathologist).

	No. of cases (%)					
	ICC score 0	IHC score 0	ICC score 1+	IHC score 1+	ICC score 2+	IHC score 2+
Original diagnosis	30 (63.9)	17 (36.9)	9 (19.1)	21 (4.7)	7 (14.9)	8 (17.4)
Revised cases (first pathologist)	26 (56.5)	15 (32.6)	16 (34.8)	26 (56.5)	4 (8.7)	5 (10.9)
Revised cases (second pathologist)	26 (56.5)	14 (30.4)	15 (32.6)	26 (56.5)	5 (1.9)	6 (13.1)

Abbreviations: ICC, immunocytochemical; IHC, immunohistochemistry.

After blinded expert revision, agreement between cytological and histological HER2 assessment improved for both pathologists (Tables 2 and 3). Concordance was higher for HER2‐low cases (IHC 1+ and 2+ without gene amplification), exceeding 90% for both observers (95.0% for the first pathologist and 92.6% for the second). In contrast, discordance was more frequently observed in cases scored as HER2 0 on cytology, which were often reclassified as HER2‐low on histological examination.

**TABLE 3:** Evaluation of agreement between original set and revised sets (first and second pathologist).

Evaluation		No. of cases (%)		
		Original diagnosis	Revised cases by first pathologist	Revised cases by second pathologist
Cytology 0	Histology 0	14 (30.4)	13 (28.1)	12 (26.1)
Cytology +1	Histology 0	3 (6.5)	1 (2.2)	2 (4.4)
Cytology +2	Histology 0	0	0	0
Cytology 0	Histology +1	12 (26.1)	12 (26.1)	13 (28.1)
Cytology +1	Histology +1	5 (10.9)	12 (26.1)	11 (23.9)
Cytology +2	Histology +1	4 (8.7)	2 (4.4)	2 (4.4)
Cytology 0	Histology +2	4 (8.7)	1 (2.2)	1 (2.2)
Cytology +1	Histology +2	1 (2.2)	3 (6.5)	2 (4.4)
Cytology +2	Histology +2	3 (6.5)	2 (4.4)	3 (6.5)

Diagnostic performance analysis confirmed these findings (Tables 4 and 5). For the first pathologist, cytological assessment of HER2‐low status achieved a sensitivity of 59.4% and a specificity of 92.9%, with a PPV of 95.0% and an NPV of 50.0%. Similar results were obtained by the second pathologist, with a sensitivity of 56.3%, a specificity of 85.7%, a PPV of 90.0%, and an NPV of 46.2%. ROC curve analysis demonstrated good diagnostic accuracy, with an AUC of 0.76 and 0.71 for the first and second pathologists, respectively (Figure 4).

**TABLE 4:** Sensitivity, specificity, PPV, and NPV in the revised set (first pathologist).

Metric value		
Sensitivity	59.38%	CI 95%, 42.26–74.48
Specificity	92.86%	CI 95%, 68.53–98.73
PPV	95%	CI 95%, 76.39–99.11
NPV	50%	CI 95%, 32.06–67.94

*Note:* HER2‐low positivity was defined as HER2 immunohistochemistry score 1+ or 2+ without gene amplification. HER2 score 0 was considered negative. Sensitivity, specificity, PPV, and NPV were calculated considering histological assessment as the reference standard.

Abbreviations: CI, confidence interval; NPV, negative predictive value; PPV, positive predictive value.

**TABLE 5:** Sensitivity, specificity, PPV, and NPV in the revised set (second pathologist).

Metric value		
Sensitivity	56.25%	CI 95%, 39.33–71.83
Specificity	85.71%	CI 95%, 60.06–95.99
PPV	90%	CI 95%, 69.90–97.21
NPV	46.15%	CI 95%, 28.76–64.54

*Note:* HER2‐low positivity was defined as HER2 immunohistochemistry score 1+ or 2+ without gene amplification. HER2 score 0 was considered negative. Sensitivity, specificity, PPV, and NPV were calculated considering histological assessment as the reference standard.

Abbreviations: CI, confidence interval; NPV, negative predictive value; PPV, positive predictive value.

### ER, PgR ICC and IHC data

ER expression in cytological and histological cases was comparable with a high Cohen's κ coefficient being obtained (κ: >0.8 for both pathologists compared with the original evaluation). PR expression in cytological and histological cases was comparable, with a moderate Cohen's κ (0.5 < κ < 0.8 for both pathologists compared with the original evaluation).

## DISCUSSION

FNAC is a relevant and important tool for the preoperative pathological evaluation of breast cancer, although it is increasingly being replaced by core biopsy. This procedure allows for a simple, rapid, cost‐effective, and relatively painless diagnosis.

According to the International Academy of Cytology Yokohama System for Reporting Breast Fine‐Needle Aspiration Biopsy Cytopathology, FNAC results are categorized as nondiagnostic, benign, atypical, suspicious for malignancy, or malignant.^18^ The reported sensitivity for carcinomas ranges from 35% to 95%, whereas specificity varies from 48% to 100% in the literature.^19^, ^20^

Regarding prognostic markers, results from CBs showed consistent, reliable performance. ER, PR, and HER2 performed similarly, although PR and HER2 had lower results. In particular, concordance between CB and histological data ranges from 90% to 98.2% for ER, 77.5% to 96% for PR, and 78% to 98% for HER2.^21^ In most studies, the concordance of HER2 evaluated in cytological samples with respect to histological samples, according to the classic CAP scores, emphasized greater attention to score 3+ compared to all others, given the critical role of targeted therapy for these patients over the past 3 decades. However, the development of new antibody‐drug conjugates has challenged the traditional reliance on HER2 expression or gene amplification in targeted therapy, introducing HER2‐low as a distinct category in breast cancer treatment. Although patients with HER2‐low expression benefit from these innovative therapies, HER2 IHC assessment reconstructs the traditional concept of HER2‐negative cases. Based on this definition, patients with HER2 IHC 1+ or 2+ and negative FISH are classified as having low HER2 expression.^22^

In particular, temporal and intratumoral heterogeneity significantly influence HER2 dynamics, mainly in HER2‐0 or HER2‐low tumors.^23^ Intratumoral heterogeneity, characterized by variable HER2 expression intensity and distribution within different regions of the same tumor, further complicates diagnosis and treatment. Filho et al.^24^ identified intratumoral HER2 heterogeneity in 10% of breast cancers, significantly affecting the accuracy of diagnosis and therapeutic efficacy. These findings underscore the need for comprehensive and dynamic HER2 assessment strategies to optimize patient outcomes. In this context, FNA could not be representative of the entire tumor. Consistent with these findings, our series—including samples with more than 100 neoplastic cells—demonstrated a high specificity and a PPV of 95%, whereas the sensitivity was moderate and the NPV was 50%. This suggests that a 1+ or 2+ positive result in cytological testing is highly indicative of HER2‐low status in histological samples. However, a 0 score in cytology does not reliably predict the same score in histological samples.

There are currently no established guidelines specifically validating HER2 testing on cytological specimens. CBs derived from FNAC can often provide sufficient material for diagnosis, given that FNAC cytology and CNB demonstrate comparable specificity and reliable clinical performance.^10^, ^25^ However, other researchers have highlighted the superior accuracy of CNB in determining histological grade and assessing ER, PgR, and HER2 status when compared to surgical samples in breast cancer cases.^17^ Indeed, biomarker detection between histological samples and smeared cytological material from breast cancer patients shows low concordance, but FNAC‐derived cell blocks demonstrate high concordance with histological samples for ER and HER2 status in the majority of cases.^9^, ^26^ However, when analyzing tumor subtype classification, FNAC‐derived cell blocks exhibit greater concordance with histological samples for HER2‐positive and triple‐negative breast cancer compared to other subtypes.^9^ Nonetheless, it is essential to recognize the potential for interobserver variability in interpreting HER2 IHC in both cytological and surgical specimens, particularly in cases near cutoff thresholds (e.g., IHC 1+ vs. IHC 2+), which are inherently more prone to subjective differences in scoring.^9^, ^27^

In our series, we focused exclusively on cases diagnosed with 0, 1+, or 2+ without HER2 gene amplification. We observed a discordance rate of 13.04% in cytology and 10.87% in histology between the original and revised assessments. This highlights the need for more precise scoring not only in histopathology but also in cytopathology, especially given the potential application of the revolutionary trastuzumab deruxtecan therapy.

A previous series of cytological cases focused on the characterization of metastases, with a special emphasis on the HER2‐low category, has been reported, showing a 19% discordance. Of the discordant cases, 50% (four of eight) showed a shift from HER2‐0 in primary tumors to HER2‐low in metastatic lesions, 37.5% (three of eight) shifted from HER2‐low in primary tumors to HER2‐0 in metastases, and 12.5% (one of eight) transitioned from HER2‐low in the primary tumor to HER2‐positive in metastatic lesions.^28^ According to these authors, the discordance is attributed to the temporal heterogeneity observed in breast cancer, characterized by a shift in HER2 status in metastases compared to primary breast carcinoma. This discordance in histological samples of primary and metastatic breast cancers has been shown to influence management strategies in up to 20% of cases, potentially providing new therapeutic options for a significant number of patients.^29^, ^30^

Our article presents several limitations. In our series, a systematic approach to assess intratumoral heterogeneity of HER2 staining was not planned; therefore, such an analysis was not performed. Thus, this represents a limitation of our study and prospective and systematic assessment of heterogeneity should be incorporated into future studies. A further element that may threaten the reliability and overall significance of these findings relates to potential errors occurring during the pre‐analytical handling of histological samples, as well as to the variability introduced by different techniques employed in CB preparation. We believe that such limitations do not diminish the value of these observations; rather, they emphasize the imperative for the cytopathology community to pursue a more rigorous and systematic exploration of this matter.^31^, ^32^ The last limitation of our study is the relatively small number of cases (*n* = 46) and the single‐institutional nature of the series, which inevitably reduces the statistical power and the external generalizability of our findings. Although our results support the diagnostic utility of FNAC‐derived cell blocks in assessing HER2‐low status, validation in larger and multi‐institutional cohorts is essential to confirm the robustness of these observations and to standardize their clinical application.

In conclusion, our study emphasizes that cytology, when compared to histology of the same sample, is a valid tool for characterizing HER2‐low cases, considering the higher reliability of cases with a score of 1+ or 2+ without gene amplification compared to cases with a score of 0. Therefore, when cytology is the only tool for the predictive characterization of primary or metastatic breast carcinomas, a score of 1+ or 2+ without gene amplification is sufficiently accurate for determining the HER2‐low status. In cases with a score of 0, it may be necessary, when possible, to continue the diagnostic process to potentially guide the patient toward treatment with trastuzumab deruxtecan.

## AUTHOR CONTRIBUTIONS

**Giuseppe D’Abbronzo**: Conceptualization; investigation; methodology; validation; software; writing—original draft; and writing—review and editing. **Stefano Lucà**: Conceptualization; investigation. **Immacolata Cozzolino**: Visualization; investigation; supervision. **Marina Accardo**: Visualization; investigation; supervision. **Carminia Della Corte**: Data curation; supervision. **Francesco Iovino**: Data curation; upervision. **Simona Parisi**: Data curation; supervision. **Ilaria Tedesco**: Data curation; supervision. **Francesco Ingallinella**: Investigation; visualization; formal analysis; data curation. **Francesca Grasso**: Data curation; supervision. **Renato Franco**: Conceptualization, methodology; investigation; supervision; writing—review and editing. **Marco Montella**: Conceptualization; methodology; investigation; supervision; formal analysis; writing—original draft; writing—review and editing.

## CONFLICT OF INTEREST STATEMENT

Carminia Della Corte reports consulting fees from Astra Zeneca, MSD, Merck, Regeneron, Lilly, Gentili, Novartis, Daichii, Roche, Amgen, Pfizer, Genmab, and Pharmamar; travel fees from Astra Zeneca, MSD, Merck, Regeneron, Lilly, Novaritis, Roche, and Amgen; and participation on a data safety monitoring board or advisory board with Astra Zeneca, MSD, Merck, Regeneron, Gentili, Novartis, Daichii, Amgen, and Pharmamar. Ilaria Tedesco reports fees for professional activities from Università degli Studi della Campania Luigi Vanvitelli. The other authors declare no conflicts of interest.

## Data Availability

The data that support the findings of this study are available from the corresponding author on reasonable request.
